# Tip of the Iceberg

**DOI:** 10.1017/ipm.2020.69

**Published:** 2020-05-28

**Authors:** Fiona Sherlock

I was drowning, of course. My throat stung from the salt and the cold as the waters filled the passageways. The impulse to inhale was too much to fight, my body was losing the war with itself.

As I lay suspended between the last swathes of sleep and the light tinkles of a waltz from the alarm clock, the panic subsided. The softness of the duvet anchored me to the present, as my breathing steadied. I had had this dream many times before, but this was the first time I had gone overboard. The sense of impending doom was now accompanied by the horrific physicality, a knife-like filling of icy water into my lungs.

Ordinarily, it heralded the nerves and excitement of an upcoming event, the presentation of a conference paper or some such. I usually made it onto a lifeboat, for wearily witty conversation enshrouded in a mink, chartreuse jelly repeating on me.

The weight of his cool hand roused me to consciousness. I was still not used to his presence this late in the morning.

‘Now, now, you’re ok’, he drew me into the sallow skin of his chest, as I continued to shiver. ‘It was just a bad dream!’

A robustness of spirit grew as I noted the warm rays of the sun streaming across the bed frame.

‘I was drowning this time, that’s never happened before’, coming back to consciousness, I noted the red flashing of my phone from the table.

‘It’s only natural, given what’s going on’, he threw on an old jumper to begin his own day’s work, the dining room given over to disorganised reams of hastily printed paper. ‘But you’re the shrink.’

Ignoring the comment, I rang through to the ward, eight missed calls did not bode well.

‘Jeannie, hi, I missed your call.’

‘It’s Mrs O’Brien, she’s back again. Dr Peters is with her now, but I thought you’d want to know’, there was silence in the usually bustling reception.

‘Patch me down there, thanks’, I swung my legs out of the bed, needing to walk off the adrenalin of the news. It had been a long time since she had been in.

‘Hi, it’s Dr Conte.’

‘Hello’, he sighed.

‘… I believe you have Simone O’Brien there.’

‘Yes her husband brought her in, well he’s out in the car …’

‘What is the matter?’

‘She is distressed, this time her fears are linked to believing there is some impending invasion by advanced reptilian creatures. She’s felt the need to barricade herself in the garage. She thinks the food is poisoned, has not eaten, She does not look well, is dishevelled. I’m going to admit her.’

‘That sounds good, keep her close to the nurses station, offer fluids, monitor obs, up her medication if necessary.’

‘Yes, Dr Conte’, he was almost robotic in his responses, but that had more to do with his own attitude, I didn’t let it bother me. ‘I’m on call tonight, I’ll keep a close eye on her.’

I set the phone back into its cradle and freshened up the bed, allowing the cold air of the morning into the intimate space of the room. Simone O’Brien had been unwell for most of her life, but something had finally settled with her a few years ago. Her medication was effective and normality had returned. There was something about her that resonated with me, despite my professional coolness. We even looked alike, Jeanie had commented. I liked her humour in lucidity, and I admired her hopefulness in the face of setbacks. It was not right to be affected by patients, but I am also human and liked the woman. It would not be possible to return to sleep yet, and hearing the sprightly cartoon theme tune, I ventured down for cuddles with my daughter.

‘You’re a bit big for Tom and Jerry aren’t you? But not too big for a hug, come on pet’, I sat into the armchair as she crawled onto my lap. Physically, of course, she was too big, but that scent of apple shampoo retained her identity as a tiny infant. The softness of her skin reminded me of that innocence, as I swept the matted curl from the collar of her pyjamas. It was more than time for homeschooling, but I drank in the warmth of her frame for a few moments longer.

‘Where’s Marta?’

‘She’s not up yet?’

A chill ran up my spine.

‘She was on the phone to El Salvador late last night. Her sister is sick’, my daughter curling her thumb to the edge of her bottom lip.

I exhaled the breath I hadn’t meant to hold on to. It was still not like the militarily organised and highly energetic Marta to still be asleep. She had never taken a lie-in during her ten years with us, preferring a dawn run or early shopping expeditions into town on her days off.

As the cartoon credits rolled, the broadcaster’s homeschooling regime began, a bust of the young enthusiastic present appearing in the bottom of the screen:

‘Welcome to school at home! This morning we’re going to start with history, so grab a glass of water and your notebook, it’s time to learn! Today is the anniversary of the sinking of the Titanic, learn all the facts after the break.’

‘Righto miss, get yourself organised, more cartoons later’, I gave one final hug, drawing in all the goodness of that contact. That hug was for me, not for her, as she prepared for the lesson.

I followed the scent of percolated coffee to the dining room, where three screens showed the world’s stock markets, red lines tumbling down. I had seen too many graphs of late and they had begun to lose all meaning.

‘Some small rallies here and there, but still niente.’

Not one to ordinarily proffer unsolicited interpretations, I realised he was playing solitaire on the small laptop screen. The deck was ready to pop, I saw a full game had been played.

‘Have you seen Marta? Penny says she’s still in bed.’

As the cards leaped diagonally across the screen, he held my gaze – that same shadow of concern flickering back at me.

‘I heard her crying when I was up in the night-time, I couldn’t sleep, came down and had to check the Nikkei. Her sister is in ICU.’

‘You don’t think …’

‘No I didn’t swab her then and there love’, annoyance creased his brow, as I left him to it, winding down to her room behind the kitchen.

It was natural to be worried, wasn’t it?

No sound behind the door, as I gently rapped. ‘Marta? Are you ok? Can I come in?’

‘Hmmm’, came the soft voice.

The air was stale, the curtains still drawn. I sat on the edge of the single bed, resting a hand on her shoulder, as she lay turned to the wall.

‘Marta, I’m so sorry your sister is sick, she is in hospital?’

Her birdlike frame shook in accord.

‘She is dying, she is dead. My son … he is all alone’, the guttural ache of a mother’s distress cracked through. ‘He does not understand.’

‘Of course. Who is he with now?’

‘No one will take him, he is at her house. Until social services come’, another huge groan.

‘Alone?’

She responded by wailing more. Marta had left him as a chubby toddler to come and work for them.

‘Oh now, we will put our heads together and think of something … Marta are you feeling ok?’ The rims of her eyes were raw, but the ruddiness extended across her face. The room was hot.

‘I am not sick, if that’s what you mean, I will get up now.’

‘No, no, take your …’

She gently pushed me aside, gathering her clothes to dress.

The shower clicked on as I poured a bowl of cereal and scrolled my phonebook for someone who could help Marta … a call to the embassy would be the best option. I hit out a text to Jeannie. ‘Has Mrs O’Brien been admitted yet?’

I needed the radio to get some perspective and space from the thoughts cloying for my attention.

The silky tones of the veteran presenter spoke gently. ‘Continuing with the greatest hits from the decade that fashion forgot, the 1980s, here’s some REM with *The End of the World as We Know It,* because we have to laugh, don’t we?’

And let me tell you, reader, I did, because I didn’t want to cry, and I was feeling far too emotional. The expulsion of nervous energy soothed me, it was almost 11, time to venture another spell of rest. It was thankfully, a thick blanket of sleep, one which I would soon become accustomed to. The physical exhaustion of the next few months pushed the White Star Line visions to recesses of my mind as deep as the ill-fated vessel herself. I was not deprived of the balm of hurt minds. Jeanie stopped wearing make-up, as the admissions grew, the community referrals ballooned. It was good to help. Progress reversed for many, bearing the weight of isolation. Of unresolved grief for that old way of life.

I now feel the hard whalebone corset compressed against the metal bars of the promenade deck. It is a warm afternoon as I squint and make out the receding headland of Cobh.

‘Morning mommy’, the trail of spilt tea across the carpet, Penny rattles a breakfast tray across the room. Droplets of tepid tea from the saucer draw me into wakefulness as she clambers under the covers. There is too much room in this bed now, and I’m glad of her body beside mine.

‘Only two more sleeps til Christmas!’ she smiles, looking at the edge of her dad’s pillow. The effort to remain cheerful is touching. A hearty baritone choir chugs out the final verse of Good King Wenceslas on the radio before two bongs herald the news.

‘Good morning, welcome to this morning’s news on December 22nd, 2020. The full extent of the mental health fallout from this year’s Coronavirus pandemic may not be known for almost a decade, according to a paper being presented this morning by Dr Amanda Conte, consultant psychiatrist at St Imelda’s Psychiatric Hospital. As the country enters its fifth month without reporting any cases of Coronavirus …’

‘Let’s turn it off for the moment, Pen.’

The room now silent, I hear Marta’s vigorous hoovering downstairs. I slurp the now cold tea, feeling the warmth of the winter sun streaming across my face. The soft skin of my child next to mine, peaceful and content in this singularly perfect moment.

